# Encapsulation of Bovine Primordial Follicles in Rigid Alginate Does Not Affect Growth Dynamics

**DOI:** 10.3390/bioengineering11070734

**Published:** 2024-07-19

**Authors:** Kathryn L. McElhinney, Erin E. Rowell, Monica M. Laronda

**Affiliations:** 1 Stanley Manne Children’s Research Institute, Ann & Robert H. Lurie Children’s Hospital of Chicago, Chicago, IL 60611, USA; kmcelhinney@luriechildrens.org (K.L.M.);; 2 Department of Surgery, Feinberg School of Medicine, Northwestern University, Chicago, IL 60611, USA; 3 Department of Pediatrics, Division of Endocrinology, Feinberg School of Medicine, Northwestern University, Chicago, IL 60611, USA; 4 Department of Obstetrics and Gynecology, Feinberg School of Medicine, Northwestern University, Chicago, IL 60611, USA

**Keywords:** bovine ovary, primordial follicle, follicle culture, bioprosthetic ovary, fertility preservation

## Abstract

The only fertility preservation and subsequent restoration option for many patients facing gonadotoxic treatments is ovarian tissue cryopreservation and transplantation. While this process is successful for some, there is significant room for improvement to extend the life of the transplant and to make it safe for patients that may have metastatic disease within their ovarian tissue. We need a deeper understanding of how the physical properties of the ovarian microenvironment may affect folliculogenesis to engineer an environment that supports isolated follicles and maintains primordial follicle quiescence. Bovine ovaries were used here as a monovulatory model of folliculogenesis to examine the effects of primordial follicle activation and growth under different physical conditions. We found that there were no differences in activation, growth or survival when primordial follicles were cultured in isolation or in situ (remaining in the tissue) under two significantly differently rigid alginate gels. To determine if the extra rigid environment did not affect activation in isolated follicles due to an immediate activation event, we used 5-ethynyl-2′-deoxyuridine (EdU) to track follicle activation during the isolation process. We identified EdU incorporation in granulosa cells after primordial follicles were isolated from the surrounding extracellular matrix (ECM). These findings support that isolation of primordial follicles from the ECM is an activating event and that the differentially rigid environments assessed here had no effect on follicle growth. Further work is needed to suppress activation in primordial follicles to maintain the ovarian reserve and extend the life of an ovarian tissue transplant.

## 1. Introduction

Premature ovarian insufficiency (POI) results from the depletion of the ovarian reserve to less than 1000 primordial follicles before the age of 40 [1,2]. There are many causes of POI, including idiopathic, genetic, autoimmune, and iatrogenic [2]. With over 85% of childhood cancer survivors living into adulthood, it has become increasingly vital to mitigate treatment-related adverse effects and enhance long-term quality of life outcomes [3,4]. Some cancer therapies, such as alkylating chemotherapies and radiation, are gonadotoxic [5,6,7,8,9,10]. These therapies can cause POI through follicular damage and accelerated depletion [6,9,10]. Other patients that are exposed to similarly gonadotoxic therapies include those that undergo stem cell transplants for the treatment of diseases such as sickle cell disease or autoimmune conditions [11,12,13].

Ovarian tissue cryopreservation (OTC) is the only pre-treatment fertility preservation option available to patients who cannot ovulate or for those whom ovarian stimulation and egg retrieval are not advisable. The OTC process includes the surgical removal of an ovary and cryopreservation of the ovarian cortex for future autotransplantation. Ovarian tissue transplantation after OTC has resulted in more than 140 reported births [14,15]. However, the success rate and functional lifespan of the transplanted tissue varies significantly, with only 20–40% of patients having a successful pregnancy after transplant and the functional duration of the transplanted tissue ranging from 2 months to 12 years [14,16]. There is a mass activation of primordial follicles, which comprise the ovarian reserve, following transplantation that is consistent across transplant clinics [17]. This is predicted to result in an 80% reduction in primordial follicles and a decreased functional lifespan of the tissue [18,19]. To quell this mass activation event and optimize current autotransplantation techniques, a better understanding of the factors that influence primordial follicle activation and quiescence is needed. 

New ovarian fertility restoration techniques are desperately needed that support long-term restoration in a safe way, especially for those that have metastatic disease [20,21]. Additionally, transgender individuals and those with differences of sexual development may undergo elective or medically necessary gonadectomy and decide to cryopreserve and save tissue that contains germ cells [11,22,23]. This population would also benefit from alternative restoration technologies [24,25]. A bioprosthetic ovary made of isolated follicles in a three-dimensional (3D) printed gelatin scaffold restored hormone production and fertility in ovariectomized mice [26]. This approach could potentially allow ovarian follicles to be isolated from any metastatic cells prior to transplantation, enhancing the safety of fertility restoration. An engineered bioprosthetic scaffold that incorporates controls for primordial follicle activation would also improve graft longevity. 

Primordial follicles, found within the cortex of the ovary, grow better when encapsulated in stiffer environments [27]. Additionally, disruption of the extracellular matrix (ECM) in murine ovaries increased primordial follicle activation, while exogenous physical pressure added to these ovaries restores primordial follicle quiescence [28]. Several labs are investigating the ECM protein composition with human ovaries and model organisms to decipher their contribution to the physical microenvironment around follicles [29,30,31,32,33]. In our study, we sought to investigate how different encapsulating rigidities would affect the quiescence and growth of isolated or in situ bovine primordial follicles. We further explored the impact of isolating primordial follicles from bovine ovarian cortical tissue to determine if the isolation process is an irreversible activation event within follicles. Our findings contribute to the understanding of primordial follicle activation and will inform the development of future fertility restoration techniques.

## 2. Materials and Methods

### 2.1. Obtaining Bovine Ovaries 

Bovine ovaries were purchased from Applied Reproductive Technology, LLC (ART, Madison, WI, USA). Cows were postpubertal, but exact age ranges for the cattle at the time of sacrifice and ovary retrieval were not available. Prior to transport, the ovaries were washed with 2% chlorohexidine gluconate diluted with distilled water followed by a series of washes with distilled water. Ovaries were then shipped at 4 °C overnight in phosphate-buffered saline (PBS) with 50 µg/mL gentamicin and were received and processed within 24 h of animal sacrifice. Bovine ovaries without clear hemorrhagic cysts or other abnormal distortions were used for our experiments.

### 2.2. Processing Bovine Ovaries for Culture

**Figure 1:**
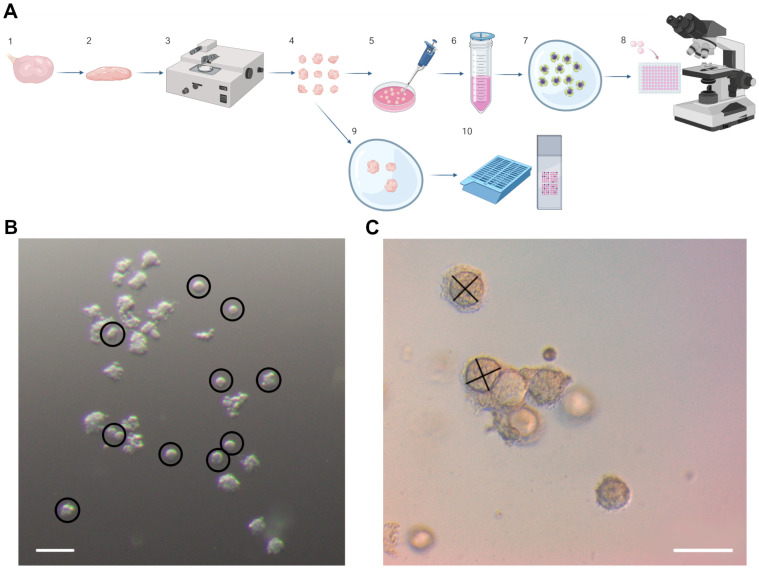
**Processing, isolation, encapsulation, and evaluation of bovine ovarian tissue and primordial follicles.** (**A**) Schematic depicting the steps taken during bovine ovarian tissue processing, follicle isolation, and follicle and tissue encapsulation: 1. Whole bovine ovaries were bisected. 2. The ovarian cortex was then separated from the medulla using a Thomas Stadie–Riggs Tissue Slicer to create 0.5 mm slices. 3. A tissue chopper then processed the slices into 1.0 × 1.0 × 0.5 mm pieces. 4. Tissue pieces then underwent an enzymatic digestion for 45 min. 5. Tissue pieces were then mechanically separated using gentle pipetting. 6. Follicles were isolated from the media using cell strainers. 7. Isolated follicles were encapsulated in alginate gel and cultured for 8 days. 8. Follicles were measured using light microscopy every other day with media changes to construct growth and survival curves. At the end of culture, follicles were fixed and analyzed using immunofluorescence. 9. Tissue pieces that did not undergo enzymatic digestion were encapsulated in alginate for in situ experiments and cultured for up to 8 days. 10. At the end of culture, tissue pieces were fixed and underwent histologic sectioning and H&E staining. (**B**) Pooled isolated primordial follicles prior to encapsulation in alginate gel. Follicles that were considered appropriate for culture based on morphologic appearance on light microscopy are circled. Scale bar is 100 µm. (**C**) To construct growth curves, the diameter of the follicle was calculated by averaging the widest diameter of the follicle with the perpendicular diameter as demonstrated by the crosshairs on the follicle in the image. Scale bar is 100 µm.

Initial processing of tissue occurred at ambient conditions and involved removing excess mesovarium and bisecting the ovary through the hilum. Tissue was further processed using a Thomas Stadie–Riggs Tissue Slicer (Thomas Scientific, Swedesboro, NJ, USA) that produces 0.5 mm thick slices (Figure 1A, step 1–2). Tissue was sliced such that the maximum amount of cortical tissue was removed from the ovary, i.e., away from the cut side of the ovary. From this point on, tissue not being actively manipulated was kept in Dulbecco’s Modified Eagle Medium (DMEM; Caisson, DFL13-6X500ML) for a limited time (approximately 5 min) at ambient temperature to maintain moisture prior to moving on to the next steps with media equilibrated in 5% CO_2_. Tissue slices were then further processed using a tissue chopper into 1 mm by 1 mm by 0.5 mm pieces (Figure 1A, step 2–4).

### 2.3. Primordial Follicle Isolation

Bovine cortical pieces were placed into an enzymatic digestion medium containing 1% bovine serum albumin (BSA), 0.08 mg/mL Liberase TL (Sigma-Aldrich, St. Louis, MO, USA), 0.2 mg/mL DNase I (Sigma-Aldrich, St. Louis, MO, USA) in DMEM. Tissue pieces in the digestion medium were then incubated on an orbital shaker for 45 min at 37 °C and 5% CO_2_. The tissue was then mechanically agitated with manual pipetting to release the follicles from the stroma into the media (Figure 1A, step 5). The sample was then passed through a 70 µm cell strainer (CELLTREAT, Pepperell, MA, USA) and then through a 20 µm cell strainer (pluriSelect, Leipzig, Germany) to select for primordial follicles (Figure 1A, step 6). A maintenance medium (15% charcoal-stripped fetal bovine serum (Sigma-Aldrich, St. Louis, MO, USA) in α-minimum essential medium (MEM) (Thermo Fisher Scientific, Waltham, MA, USA) that was allowed to equilibrate at 37 °C and 5% CO_2_ for 30 min was then used to elute follicles from the strainer into a 35 mm petri dish. Follicles were then allowed to recover in the incubator for 30 min.

### 2.4. Primordial Follicle Encapsulation and Culture

Morphologically intact follicles measuring 20–40 microns were selected from the pooled follicles using a stripper pipette (Figure 1B). Alginate was previously prepared in 1% or 5% solutions (Sigma A1603) in PBS until dissolved and were used for encapsulation. Groups of 5–24 (target of 10) follicles were transferred into 5 µL droplets of alginate on parafilm squares (Figure 1A, step 7). Parafilm squares were then inverted into calcium sulfate crosslinking solution (50 mM CaSO_4_ and 140 mM NaCl) [34]. After 2 min, the alginate bead was then removed from the crosslinking solution and transferred with forceps to a prepared 96-well plate (Thermo Fisher Scientific, Waltham, MA, USA) with 100 µL of equilibrated growth medium (α-MEM containing 1 mg/mL fetuin (Sigma-Aldrich, St. Louis, MO, USA), 0.3% BSA, 5 μm insulin, 5 μg/mL transferrin, 5 ng/mL selenium (Corning Life Sciences, Tewksbury, MA, USA), 10 mIU/mL Gonal F (EMD Serono, Darmstadt, Germany)) in each well. Follicles were maintained at 37 °C throughout isolation, encapsulation, and culture. Cultures were maintained at 37 °C in 5% CO_2_ for 8 days. Half of the culture medium (50 µL) was exchanged for fresh growth medium every other day. Beads were imaged with light microscopy using a Zeiss light microscope (Zeiss Microscopy, Jena, Germany) using 4×, 10×, and 20× objectives during media changes (Figure 1A, step 8). Follicle diameters were measured using ImageJ [35]. Follicle diameter was measured as an average between the greatest diameter of the follicle and the perpendicular diameter to construct growth curves (Figure 1C). Follicles were determined to be dead if there was a decrease in size or if there was loss of the oocyte on light microscopy. At the end of culture, follicles were fixed in alginate beads using a fixative solution of 1× PBS containing 3.8% paraformaldehyde and 0.1% Triton X-100 for 30 min. Fixed alginate beads containing follicles were then held in an immunocytochemistry blocking (ICC) buffer of 1× PBS containing 0.01% Tween 20, 0.01% sodium azide, and 0.3% BSA until further processing.

### 2.5. Tissue Encapsulation and Culture

Other bovine cortical tissue pieces (1 × 1 × 0.5 mm) were encapsulated directly into alginate gel. Three tissue pieces reserved after tissue processing and prior to enzymatic digestion were placed into a 200 µL droplet of previously made 1% or 5% alginate gels (Figure 1A, step 9). Additionally, one set of experiments was conducted where tissue was maintained in culture without being encapsulated in any gel. Droplets were crosslinked in a calcium sulfate crosslinking solution as previously described for 5 min to ensure complete crosslinking of the larger droplet. Alginate beads containing tissue were transferred to a prepared 96-well plate with 100 µL of equilibrated growth medium in each well. Cultures were maintained at 37 °C in 5% CO_2_ for up to 8 days. Half of the culture medium (50 µL) was exchanged for fresh growth medium every other day. At the end of culture, tissue was fixed in Modified Davidson’s Fixative (MDF; Electron Microscopy Sciences, Hatfield, PA, USA) for 1 h and then serially dehydrated in 2 washes each of 50% ethanol, 60% ethanol, and 70% ethanol. Tissue pieces and alginate beads were then stored in 70% ethanol prior to tissue sectioning and staining (Figure 1A, step 10).

### 2.6. Tissue Sectioning and Staining

Fixed and dehydrated tissue underwent routine processing and was embedded in paraffin. Embedded specimens were then sectioned into 5 µm sections with every fifth section undergoing H&E staining. Tissue processing, embedding, sectioning, and staining was performed by the Microscopy and Histology Group at Stanley Manne Children’s Research Institute. H&E slides were digitally imaged using a 40× objective on a Hamamatsu Nanozoomer (Hamamatsu, Hamamatsu, Japan). All H&E slides from in situ tissue culture were analyzed. Follicles were categorized as primordial or not primordial based on established morphological criteria [36]. Criteria for counting included follicles with a clearly visible oocyte in the center, which may or may not include visible nuclear material. Tissue area was calculated for each sample and each condition using ImageJ, version 1.53u [35].

### 2.7. Immunofluorescence of Whole Mount Primordial Follicles

Follicles that were previously fixed and held in ICC buffer were permeabilized in 1× PBS containing 0.3% BSA and 0.01% Triton X-100. Follicles were then washed in ICC buffer and incubated with a 1:500 dilution of anti-MSY2 or anti-DDX4 for 16–18 h at 4 °C. The primary antibody was detected using Alexa Fluor 568-conjugated donkey anti-rabbit secondary antibody. Follicles were simultaneously stained for F-actin with Alex Fluor 488-conjugted phalloidin at room temperature for 1 h. Follicles were washed again in ICC buffer and resuspended in a 1:2000 dilution of Hoechst dye on a coverslip petri dish. Images were captured using a Zeiss LSM 800 laser scanning confocal microscope (Zeiss Microscopy, Jena, Germany) using a 40× water immersion objective. Images were processed using Zeiss ZEN software, version 2.6 (Blue Edition) (Zeiss Microscopy, Jena, Germany).

### 2.8. Atomic Force Microscopy of Alginate Gels Crosslinked in Calcium Sulfate Solution

Atomic force microscopy (AFM) was carried out on engineered materials using a Piuma Nanoindenter (Optics 11 Life, Amsterdam, The Netherlands) with accompanying software (V3.2.0). Prior to testing, 200 µL of the appropriate concentration of alginate gel (1–5 *w*/*v*%) was pipetted into the center of a 35 mm petri dish. The alginate gel was then gently encircled and submerged completely in a calcium sulfate crosslinking solution (50 mM CaCl_2_ and 140 mM NaCl) and allowed to crosslink for 20 h. After 20 h, the calcium sulfate crosslinking solution was removed from the petri dish and the crosslinked alginate gel was secured to the petri dish using PELCO Pro CA44 Instant Tissue Adhesive (Thermo Fisher Scientific, Waltham, MA, USA) and submerged in PBS without calcium or magnesium to prevent the gel from drying out and any further crosslinking. Analyses for all materials were carried out using a probe manufactured by Optics 11 with a rigidity of 0.25–0.52 N/m and a tip radius of 26.5–30.0 µM. The AFM probe was calibrated in PBS to remove background noise from the liquid interface and was calibrated against a 35 mm plastic dish as per the manufacturer’s instructions. Piuma software was used to find the surface of materials and then to acquire data. Data acquisition was carried out using a three-step protocol (approach, hold, retraction) with the following settings: approach/retraction rate of 5.00 µM/s with a target value of 150 µm, and a hold time of 5 s.

### 2.9. EdU Activation Assay and Labeling

5-Ethynyl-2′-Deoxyuridine (EdU) (Thermo Fisher Scientific, Waltham, MA, USA) was utilized to label mitotically active cells in ovarian tissue during ovarian tissue processing and follicle isolation. Ovarian tissue was processed as previously described, but samples of ovarian tissue were reserved at specific time points during processing for EdU labeling to evaluate follicle activation. After the ovary was bisected, one half of the cortex removed with the Stadie–Riggs tissue slicer was processed further with the tissue chopper and the other half was incubated in DMEM containing 10 µM EdU for 1 h at room temperature. A portion of tissue was reserved after being processed by the tissue chopper and similarly incubated in DMEM containing EdU for 1 h. The remaining tissue was enzymatically digested as described previously. After enzymatic digestion, another portion of tissue was incubated in DMEM containing EdU for 1 h. After incubation, all tissue was then fixed in MDF at room temperature for 1 h and then dehydrated in 2 washes each of 50% ethanol, 60% ethanol, and 70% ethanol. Tissue was then held in 70% ethanol prior to being submitted for routine sectioning and staining by the Microscopy and Histology Group at Stanley Manne Children’s Research Institute as described previously. The remaining tissue proceeded through follicle isolation. All follicles were held in maintenance medium and growth medium that contained 10 µM EdU. Approximately 1 h after follicles were isolated, a selection of follicles was then fixed in 1× PBS containing 3.8% paraformaldehyde and 0.1% Triton X-100 for 30 min. These follicles were then held in ICC buffer until further processing. The remaining follicles were then placed into a prepared round-bottom 96-well plate containing growth medium with 10 µM EdU and cultured for 1 day. Follicles were then fixed and held in ICC buffer as described previously.

### 2.10. EdU Click-iT Reaction and Immunofluorescence of Key Proteins

EdU incorporation into DNA was detected using the Click iT EdU Alexa Fluor Imaging kit (Thermo Fisher Scientific, Waltham, MA, USA) per the manufacturer’s protocol. In short, histology sides were deparaffinized and washed in a 3% BSA in PBS solution. The tissue and follicles were then permeabilized in a 0.5% Triton X-100 solution in PBS. Tissue was permeabilized for 25 min; follicles were permeabilized for 20 min. Samples were again washed with 3% BSA in PBS solution per the protocol. Samples were then incubated in the Click-it reaction cocktail for 30 min at room temperature. Samples were again washed in 3% BSA in PBS. Tissue and follicles were then stained additionally with either anti-MSY2 or anti-DDX4 and detected using Alexa Fluor 568-conjugated donkey anti-rabbit secondary antibody as described previously. Slides were mounted using Fluoroshield Histology Mounting Medium with DAPI (Sigma-Aldrich, St. Louis, MO, USA). Follicles were suspended in a 1:2000 dilution of Hoechst dye on a coverslip petri dish. Images were obtained using a Keyence BZ-X710 All-in-One inverted fluorescence phase contrast microscope (Keyence, Osaka, Japan) with 4×, 10×, 20×, and 40× objectives. Images were processed with the accompanying software (V1.3.1.1).

### 2.11. Statistical Analysis

Analysis was conducted using Prism 9 (GraphPad, San Diego, CA, USA) and R Statistical Software (v4.3.3; R Core Team 2024, Vienna, Austria). The mean ± standard error of the mean is reported and 2-way ANOVA using Bonferroni correction was used for multiple comparisons. Pairwise *t*-tests were used for post-hoc analysis. Normality of the data was confirmed using quantile-quantile plots. A multivariable regression was used to examine factors affecting follicle growth. Statistical significance was determined using α = 0.05.

## 3. Results

### 3.1. Growth and Survival of Primordial Follicles in Rigid Environments

**Figure 2:**
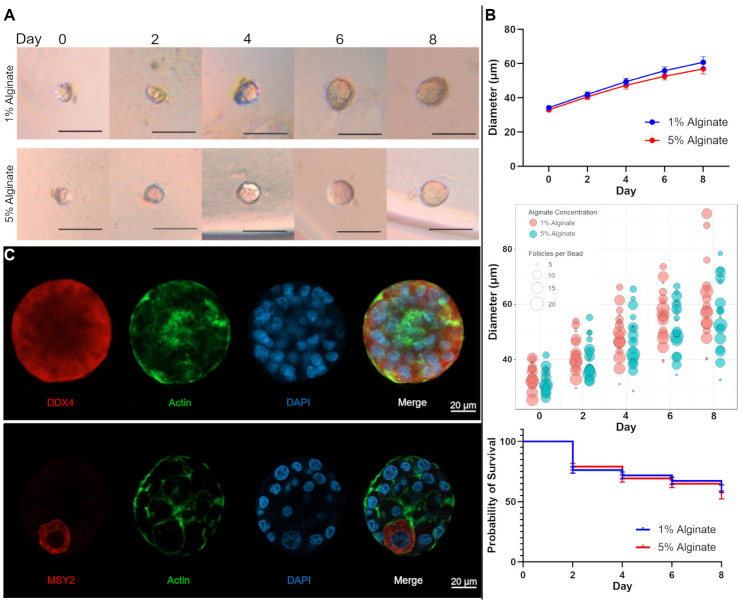
**Growth and survival of primordial follicles encapsulated in differently rigid alginate gels.** (**A**) Primordial follicles encapsulated in 1% and 5% alginate were examined under light microscopy every other day for all 8 days of culture. The same representative follicle is shown over time from each culture condition. Scale bars are 100 µm. (**B**) Growth curves were plotted using mean ± SEM of primordial follicle diameter. A multivariable regression utilizing day, number of follicles encapsulated per bead, and alginate concentration was constructed to analyze the effects of these covariates on follicle diameter. Survival curves between the two concentrations of alginate were also constructed. (**C**) Whole-mount immunofluorescence for proteins DDX4 and MSY2, phalloidin (actin), and DNA (DAPI) was performed on bovine follicles cultured for 8 days.

Primordial follicles were isolated from bovine ovaries and cultured in alginate gels to determine if the percentage of alginate, or the rigidity of the environment, would affect their overall growth and survival (Figure 2A). To obtain an alginate that is within the desired rigidity of our target to mimic the bovine ovarian cortex, we measured the Young’s modulus of 1–5% alginate crosslinked with calcium sulfate (Figure S1) [34]. Based on these results, we proceeded with 1 and 5% for our cultures. A median of 10 follicles (range 5–24) were encapsulated per bead in either 1% or 5% alginate across five experiments. There were 38 beads total, 19 in each condition (Figure S2). The mean diameter of follicles encapsulated in 1% alginate was 34.2 ± 1.0 µm on day 0 and increased to 60.8 ± 3.2 µm after 8 days in culture. The diameter of follicles encapsulated in 5% alginate was 33.0 ± 0.9 µm and increased to 56.9 ± 2.9 µm after 8 days in culture. The difference in growth between the 1% alginate and 5% alginate conditions was not significant (*p* = 0.07; Figure 2B). After 8 days in culture, 60.8 ± 3.1% of follicles survived in the 1% alginate encapsulation versus 55.6 ± 3.3% that survived in the 5% alginate encapsulation (Figure 2B). Follicles cultured in 1% and 5% alginate appeared morphologically similar at the end of 8 days in culture (Figure S3). To better understand the influence of different factors on follicle growth in in vitro culture, we constructed a multivariable regression (Figure 2B). We selected day, number of follicles encapsulated per alginate bead, and alginate concentration of encapsulation as covariates. A progression of 1 day was associated with a significant increase in follicle diameter (β = 3.17 µm/day, *p* < 0.001). The number of follicles encapsulated per alginate bead was inversely associated with follicle diameter (β = –0.29 µm/follicle, *p* = 0.02). A one-unit increase in alginate concentration was associated with a 0.67 µm decrease in follicle diameter (β = −0.67, *p* = 0.04). When assessing the covariates independently, only days in culture remained a significant contributor in the linear model (β = 3.17 µm/cm, *p* < 0.001). Both the number of follicles encapsulated per bead (β = −0.27 µm/cm, *p* = 0.11) and the concentration of alginate of encapsulation (β = −0.62 µm/cm, *p* = 0.18) failed to maintain significance when analyzed independently. To verify the presence of intact bovine follicles at the end of culture, follicles were fixed and then analyzed using immunofluorescence with staining specific for markers of oocytes, DDX4 and MSY2 [37,38,39]. Actin and nuclear co-staining was used to evaluate the integrity of the follicle. Follicles stained for both DDX4 and MSY2 and appeared to have a single layer of granulosa cells surrounding the oocyte (Figure 2C). A representative image of a follicle with an asymmetrical oocyte is also shown. Actin appropriately surrounded the follicle, suggesting an intact structure. The pattern of staining we examined here with DDX4 and MSY2 is similar to what has been previously reported in mouse and rhesus macaque follicles [27,40]. After we did not find a difference in growth or survival of isolated primordial follicles grown in vitro, we hypothesized that culturing primordial follicles encapsulated in a more rigid alginate gel in situ would result in an increased number of primordial follicles over 8 days of culture.

### 3.2. Survival of Follicles Encapsulated in Alginate In Situ

**Figure 3:**
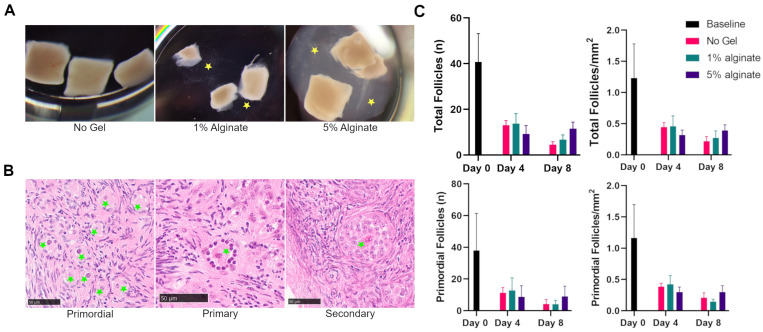
**Evaluating survival of follicles grown in situ in differentially rigid conditions.** (**A**) Tissue was cultured for up to 8 days in either no gel, 1% alginate, or 5% alginate. Alginate is marked with yellow stars. (**B**) Representative images of follicles found in tissue that was cultured for up to 8 days. Primordial, primary, and secondary follicles are noted by green stars. Scale bars are 50 µm. (**C**) Total and primordial follicles were counted in tissues fixed on days 0, 4, and 8 in cultures without gel, in 1% alginate, and in 5% alginate. The mean ± SEM are graphed for each condition and timepoint and represented as total number of all follicles or primordial follicles (**left**) or as follicles per area (**right**).

A total of four replicates containing three pieces of ovarian cortex each were analyzed for each condition. Analyses included a baseline measurement on day 0 of tissue that was not encapsulated in any gel, and tissue on days 4 and 8 that were cultured either without alginate gel, in 1% alginate gel, or in 5% alginate gel (Figure 3A). A total of 84 pieces of bovine ovarian cortex were serially sectioned and every fifth section was analyzed (Figure 3B). At baseline, 40.8 ± 12.4 total follicles were observed within the tissue (Figure 3C). This declined in all conditions across the 8 days. The total number of follicles in tissue not encapsulated in gel decreased to 4.5 ± 1.3 follicles after 8 days in culture. Similarly, the total number of follicles in tissue encapsulated in 1% alginate decreased from baseline to 6.8 ± 2.1 follicles. The total number of follicles in tissue encapsulated in 5% alginate decreased to 11.5 ± 2.9 follicles after 8 days in culture. This trend was consistent when only primordial follicles were examined, and when the surface area of the specimen was accounted for (Figure 3C). While the number of primordial follicles and number of total follicles was greater in the 5% alginate condition after 8 days in culture, these numbers were not significantly different across the conditions (*p* = 0.99). Post hoc analysis similarly confirmed that there was no significant difference between the number of follicles (no gel vs. 1% alginate, *p* = 1.0; 1% alginate vs. 5% alginate, *p* = 0.42; no gel vs. 5% alginate, *p* = 0.36). Because there was no significant difference in follicle numbers across groups, we hypothesized that disrupting the ovarian cortex when the tissue is processed may cause activation of primordial follicles and subsequent decline in total numbers.

### 3.3. Activation of Primordial Follicles during Isolation and Encapsulation

**Figure 4:**
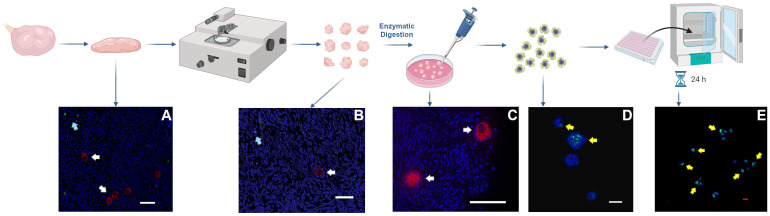
**Incorporation of EdU during tissue processing for primordial follicle isolation.** Bovine cortex tissue samples were reserved at specific points during ovarian tissue processing and primordial follicle isolation and incubated with EdU. Tissue in panel (**A**) was incubated with EdU immediately after the ovarian cortex was removed using the tissue slicer. Tissue in panel (**B**) was incubated immediately after processing by the tissue chopper. Tissue in panel (**C**) was incubated after enzymatic digestion. Images in (**A**–**C**) represent sections probed for EdU (green) and MSY2 (red; white arrows). Isolated follicles in panel (**D**) were incubated immediately after mechanical isolation, and follicles in panel (**E**) were cultured with EdU for 1 day. Stromal cells, but not follicles, were EdU-positive (green, blue arrows) in the tissue. Isolated follicles in (**D**,**E**) contained EdU-positive granulosa cells (yellow arrows). White scale bars are 50 µm and the red scale bar is 100 µm.

To assess follicle activation during ovarian tissue processing and isolation, we opted to repeat our follicle isolation procedure and incubated tissue and follicles with EdU at specific time points. EdU incorporates into replicating DNA and is a marker of mitotic activity during S phase, and any incorporation of EdU into granulosa cells would indicate an activation of primordial to primary follicles or proliferation of granulosa cells within follicles of subsequent stages [41,42]. Three biological replicates (ovaries from different cows) were examined. In all three experiments, there was evidence of mitotic activity within the granulosa cells surrounding primordial follicles within freshly isolated follicles and follicles after 1 day of culture (Figure 4). EdU was not observed in the cells surrounding primordial follicles within tissue slices, tissue after being processed by the tissue chopper, or in tissue after enzymatic digestion. EdU was incorporated into stromal cells away from DDX4- and MSY2-stained primordial follicles, which suggested that EdU was taken up by cells within the tissue and that the Click iT reaction was successful.

## 4. Discussion

There is a pressing need to improve upon the current techniques used for ovarian restoration. The mass activation of primordial follicles following ovarian tissue transplantation leads to a truncated tissue lifespan. The risk of reimplanting malignant cells also limits the number of patients who are candidates for this procedure [18,19,20,21]. The development of a bioprosthetic ovary holds the potential for optimizing the microenvironment surrounding primordial follicles, thus preventing premature activation and loss, while also allowing for the removal of metastatic disease [26,43]. However, the physical and biochemical properties of the ovarian matrisome need to be better understood to inform a competent scaffold for a bioprosthetic ovary. To contribute to this knowledge, we examined the impact of encapsulating isolated bovine primordial follicles in alginate gels of differing rigidities on activation, growth, and survival.

In this study, we cultured bovine primordial follicles in a 3D alginate system for 8 days. To our knowledge, this is the first report of successful in vitro culture of isolated bovine primordial and/or primary follicles [44]. We confirmed the presence of intact follicles at the end of culture by immunofluorescence [37,38,39].

We hypothesized that a more rigid environment would result in decreased activation and growth in primordial follicles based on prior work that had shown more rigid environments resulted in improved ovarian follicle integrity when cultured in vitro and that exogenous pressure resulted in maintenance of primordial follicle quiescence [27,28]. While a one-unit increase in alginate concentration was associated with a small decrease in follicle diameter, the concentration of alginate failed to maintain significance when analyzed independently. This finding sharply deviates from our original hypothesis, which was based on prior work by Nagamatsu et al., who reported that applying 33.3 kPa of exogenous pressure to murine ovaries maintains primordial follicle dormancy [28]. Several crucial differences in our study could explain these unexpected findings. First and foremost, our study was performed using bovine follicles, which may have different follicle activation and growth dynamics from murine follicles [45,46,47,48,49]. Additionally, our study focused on isolated primordial follicles, while Nagamatsu et al. used an enzymatic treatment to denature ECM surrounding primordial follicles before applying exogenous pressure. The follicles remained in situ and were exposed to the matrisome throughout their experiment [28]. This ongoing biochemical interaction with the surrounding ECM during the application of exogenous pressure may be crucial in maintaining follicle quiescence. As our follicles were completely isolated and encapsulated by alginate, the rigidity of the surrounding environment, even if it was sufficient to maintain follicle dormancy in situ, may not have been sufficient without biochemical cues. Therefore, we immediately addressed one of these possibilities by encapsulating the cortical tissue in 1% and 5% alginate to maintain those biochemical cues. While we did observe a greater number of primordial and total follicles in the tissue cultured in 5% alginate for 8 days over control and tissue cultured in 1% alginate, the lack of observed significant differences may potentially be attributed to the rigidity of the selected alginate gels. Previous studies have shown that soft tissues generally have elastic moduli of approximately 10 kPa [50,51]. The alginate gels used in our study ranged from 20.5 ± 7.0 kPa to 271.2 ± 86.4 kPa by AFM with nanoindentation. This range could potentially exceed a threshold rigidity, beyond which a difference would not be observable. Therefore, it might be beneficial to repeat these experiments using alginate gels with lower concentrations or a wider range of rigidities.

Bovine primordial follicles are challenging to isolate and encapsulate in alginate beads, and this often resulted in a varying number of follicles per bead. Hornick et al. previously reported increased murine follicle survival and growth when primary follicles were cultured in five- or ten-follicle beads in comparison to single-follicle beads [52]. The data reported here did not compare single-follicle beads with five-and ten-follicle beads, but did reveal that the number of bovine follicles encapsulated per alginate bead was inversely associated with follicle diameter. However, this comparison was not significant when analyzed independently. Therefore, five bovine follicles within a single bead may be sufficient, but, as with murine follicles, ten-follicle beads may be superior in supporting growth for bovine follicles.

We further sought to determine whether the act of isolating primordial follicles from the bovine ovarian cortex triggers an irreversible activation event. Growth of primordial follicles from rhesus macaques cultured in vitro has been observed previously and was hypothesized to be the result of spontaneous activation [27]. To better understand if and when follicles were activated, we used EdU uptake to identify any initial proliferation event within granulosa cells during the ovarian tissue process and primordial follicle isolation process. There was increased mitotic activity in the granulosa cells surrounding primordial follicles immediately upon isolation from the tissue and increased over 24 h. This supports the previously proposed hypothesis that the isolation of primordial follicles from the ECM could induce activation.

These results carry several implications. First, they support prior hypotheses that isolating primordial follicles for growth and maturation may not be advantageous over previously described two-step methods that involve culture of primordial follicles in situ prior to maturation [53]. Clinically, our results support that disruption of the ovarian cortex by performing an ovarian biopsy or partial oophorectomy my result in primordial follicle activation, similar to what has been observed during drilling procedures for polycystic ovarian syndrome [54]. For patients in whom the goal is fertility preservation and maximal salvage of the ovarian reserve, total unilateral oophorectomy and preservation of as much intact ovarian cortex as possible may be advisable over wedge resection of the ovary or partial oophorectomy. Further study is needed to examine what biochemical and physical interactions are necessary to maintain primordial follicle quiescence during activation. Several pathways have been identified as important in primordial follicle activation [55]. While no single intervention for preventing primordial follicle activation has been identified, additional studies into the mechanisms that are activated during this follicle isolation could inform future technologies or small molecules that prevent activation during this process and could support new research that could benefit patients with POI or who have undergone OTC.

## 5. Conclusions

A deeper understanding of the physical and biochemical properties of the microenvironment surrounding the primordial follicles and how it influences activation and growth is essential. Our study demonstrates that encapsulation in a rigid environment alone is not sufficient to prevent bovine primordial follicle activation or restrict growth. Using an EdU uptake assay, we identified that granulosa cells undergo mitosis immediately after isolation from the cortical tissue. Further investigation is needed to define the mechanisms involved in maintaining primordial follicle activation or maintaining quiescence.

## Data Availability

Data are available upon appropriate request to the authors.

## Supplementary Materials

The following supporting information can be downloaded at: https://www.mdpi.com/article/10.3390/bioengineering11070734/s1, Figure S1: Rigidity of alginate gels crosslinked in calcium sulfate solution, Figure S2: Mean diameter of follicles per each bead measured over each day captured. Figure S3: Additional representative follicles from day 8 of culture in 1% and 5% alginate.
