# Supplementary material for: Encapsulation of Bovine Primordial Follicles in Rigid Alginate Does Not Affect Growth Dynamics
Source: Bioengineering (Basel). 2024 Jul 19;11(7):734. doi: 10.3390/bioengineering11070734 (PMC11273846; doi:10.3390/bioengineering11070734)
Supplement: Supplementary file 1 [file bioengineering-11-00734-s001.zip › bioengineering-3047540-supplementary.pdf]

bioengineering

MDPI

Supplementary Materials

Encapsulation of Bovine Primordial Follicles in Rigid Alginate Does Not Affect Growth Dynamics

Kathryn L. McElhinney [1,2,3], Erin E. Rowell [1,2] and Monica M. Laronda [1,3,4,*]

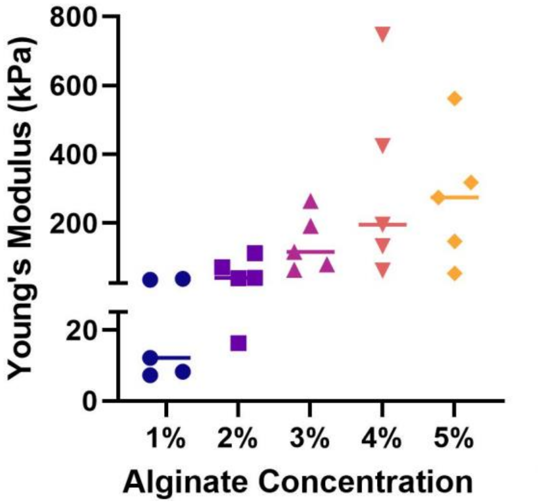

Figure S1. Rigidity of Alginate Gels Crosslinked in a Calcium Sulfate Solution. Atomic force microscopy with nanoindentation was used to evaluate the rigidity of different concentrations of alginate gels crosslinked in calcium sulfate solution. Five replicates were tested. The Young's Modulus of 5% alginate was determined to be significantly higher than 1% alginate on post hoc analysis (p=0.003).

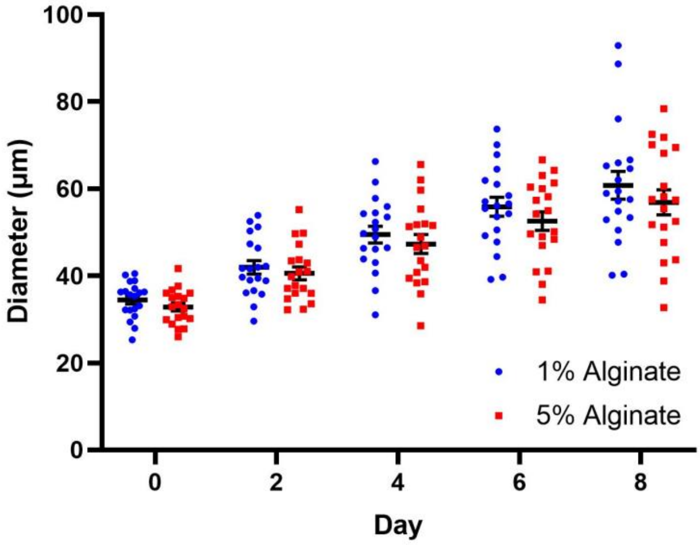

Figure S2. Mean diameter of follicles per each bead measured over each day captured. A total of 19 beads in each condition were cultured for 8 days. The mean ± SEM is graphed for each condition on each day.

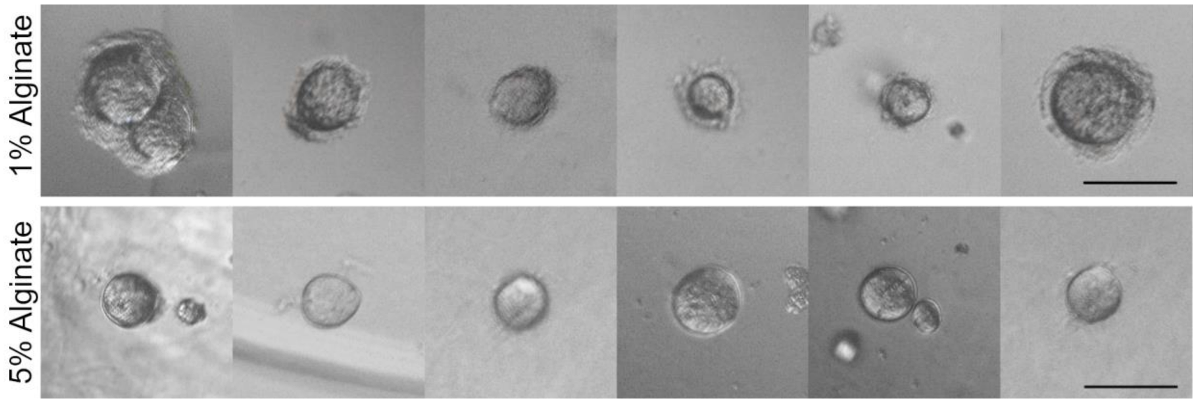

Figure S3. Additional representative follicles from day 8 of culture in 1% and 5% alginate. Follicles were morphologically similar at the end of culture in both conditions. Scale bars are 100μm.
